# Gelatinization or Pasting? The Impact of Different Temperature Levels on the Saccharification Efficiency of Barley Malt Starch

**DOI:** 10.3390/foods10081733

**Published:** 2021-07-27

**Authors:** Michael Rittenauer, Stefan Gladis, Martina Gastl, Thomas Becker

**Affiliations:** Chair of Brewing and Beverage Technology, Technical University of Munich, Weihenstephaner Steig 20, 85354 Freising, Germany; Michael.Rittenauer@tum.de (M.R.); gladisst@gmail.com (S.G.); TB@tum.de (T.B.)

**Keywords:** mashing, saccharification, sugar yield, viscometry, hydrolysis, starch granulometry

## Abstract

Efficient enzymatic hydrolysis of cereal starches requires a proper hydrothermal pre-treatment. For malted barley, however, the exact initial temperature is presently unknown. Therefore, samples were micro-mashed according to accurately determined gelatinization and pasting temperatures. The impact on starch morphology, mash viscometry and sugar yields was recorded in the presence and absence of an amylase inhibitor to differentiate between morphological and enzymatic effects. Mashing at gelatinization onset temperatures (54.5–57.1 °C) led to negligible morphological and viscometric changes, whereas mashing at pasting onset temperatures (57.5–59.8 °C) induced significant starch granule swelling and degradation resulting in increased sugar yields (61.7% of upper reference limit). Complete hydrolysis of A-type and partial hydrolysis of B-type granules was achieved within only 10 min of mashing at higher temperatures (61.4–64.5 °C), resulting in a sugar yield of 97.5% as compared to the reference laboratory method mashing procedure (65 °C for 60 min). The results indicate that the beginning of starch pasting was correctly identified and point out the potential of an adapted process temperature control.

## 1. Introduction

Malted barley is predominantly used in the brewing and distilling industry as a combined source of starch and amylolytic enzymes to produce ethanol. To obtain fermentable sugars (i.e., glucose, fructose, sucrose, maltose and maltotriose), the surface of the contained starch granules needs to be increased. This is achieved by a two-stage process eventually allowing for their hydrolysis by native amylases [1,2,3]. In a first step, the barley malt grains are mechanically crushed (milling) to increase the macroscopic surface and thus, to facilitate water absorption. Secondly, the resulting grist is blended with tempered water (mashing) to induce swelling and partial disruption of the microscopic starch granules as well as further increase the contact surface for enzymatic hydrolysis. This transformation occurs in the presence of excess water at a characteristic temperature, which is referred to as either gelatinization (GT) or pasting temperature (PT). Often, these two terms are used interchangeably [4] although they describe two different stages in the hydration and swelling of starch granules:

Gelatinization describes the melting of pseudo-crystalline regions of amylopectin [5], which is accompanied by limited amylose leaching [6] and the loss of microscopic birefringence, which can be visualized by polarized light microscopy [4]. Commonly, gelatinization characteristics of cereals are determined by means of differential scanning calorimetry (DSC). In this regard, it is important to mention that accurate measurements require samples without amylolytic activity [7,8,9,10]. As barley malt exhibits a high potential of amylases, a non-invasive starch purification method is mandatory for successive analyses [11,12]. This in turn allows for accurate DSC analyses, capable of segregating amylases from starch granules without significantly altering the native starch properties. 

Pasting summarizes continuing starch transformations occurring at temperatures exceeding the gelatinization temperature. It includes intensive swelling and total disruption of the starch granules [13] in combination with considerable amylose leaching [6,14]. The onset of pasting is characterized by an abrupt increase in viscosity, which can be determined viscometrically [15]. Optimized analytical parameters for barley malt were determined and validated in a previous study based on a Rapid-Visco-Analyzer [16]. Further investigations indicated that the pasting temperatures determined by the Rapid-Visco-Analyzer are overestimated [17]. To consider this discrepancy, arising mainly from the indirect temperature measurement principle, a compensation formula was developed to calculate the actual sample temperature at the beginning of the pasting process. This was based on the read-out sensor data [17]. 

Generally, gelatinization and pasting temperatures vary among cereals, as they depend on the properties of the starch of the respective raw material [18,19,20], the lipid concentration [21,22] and protein interactions [23,24]. Besides genetic endowments, extrinsic factors like growth conditions influence the raw material’s properties and thus, gelatinization and pasting temperatures [25,26]. This variability can be compensated for by adapting the applied process temperatures during mashing, accordingly. However, this approach can only be used within certain limits. This is because enzymes like limit dextrinase [27] and β-amylase [28,29,30,31], which are crucial for saccharification, are thermally unstable.

Based on this, it was proposed that the optimal initial mashing temperature should be as high as necessary to ensure quick starch accessibility, but concurrently, as low as possible to preserve the activity of thermolabile enzymes as long as possible. Up to now this optimal process temperature, considering fluctuating raw material properties, has been unknown and therefore was not applied in breweries and distilleries. By contrast, industrial mashing is performed according to static temperature-time regimes mostly consisting of multiple isothermal plateaus (e.g., ϑ_1_ = 62 °C, ϑ_2_ = 68 °C, ϑ_3_ = 72 °C, ϑ_4_ = 78 °C) [32]. If batches with increased pasting properties (>ϑ_1_) are processed without adaption of the initial mashing temperature, this results in reduced sugar and alcohol yields and potentially filtration problems. 

To close this knowledge gap and contribute to a better process control, malt-specific temperature levels were used as initial mashing temperature and the impact on starch morphology and subsequent enzymatic hydrolysis was investigated in this study. 

Therefore, firstly, gelatinization and pasting onset temperatures of a representative number of modern spring barley varieties were determined and secondly, isothermally applied during micro-mashing trials. The impact on starch morphology, particle size distribution, viscometric behavior as well as the production of fermentable sugars was investigated in the presence and absence of amylolytic inhibitors, allowing us to differentiate between morphological and enzymatic effects.

## 2. Materials

### 2.1. Materials and Sample Preparation

For this study, samples of 13 modern spring barley varieties (*Hordeum vulgare* L., i.e., Eileen, Gladiator, Grenada, Marthe, Nokia, Paustian, Planet, Prada, Rheingold, Soulmate, Tango, Uta and Ventina) grown in six different regions in Germany were used (*N* = 78). The samples were micro-malted according to the standardized procedure described in the method collection of the Mitteleuropäische Brautechnische Analysenkommision (R-110.00.008, MEBAK, [33]). In short, the samples were steeped for two days (day 1: 5 h wet/19 h dry, day 2: 4 h wet/20 h dry), followed by three days of germination and one day of kilning. The air and water temperature during steeping and germination phases was set to 14 °C and the relative air humidity to 95%. The final degree of steeping was 45%. Kilning was performed at 50 °C for 16 h followed by 1 h at 60 °C, 1 h at 70 °C and 5 h at 80 °C. Before further analyses, rootlets and sprouts were removed. Malted grains were dry milled using a laboratory mill (LM 3100, Perten Instruments, a Perkin-Elmer Company, Waltham, MA, USA) and the moisture content of the malt flour was determined gravimetrically according to MEBAK guidelines [33].

### 2.2. Determination of Gelatinization Temperatures

Gelatinization onset temperatures (GT_O_) were determined by differential scanning calorimetry (DSC). To ensure accurate results, each malt sample was purified in duplicate to obtain starch free of amylases [11]. Isolated starch was mixed with deionized water (ratio 1:6 *w/w*) in an Eppendorf-tube and homogenized using a vortex mixer for 60 s. This ratio was chosen as it reflects the overall starch concentration of a mash, featuring a malt-to-water ratio of 1:4 *w/w*. Immediately after homogenization, 25 µL of the suspension were transferred into a 40 µL aluminum DSC-pan, which was sealed, placed in the DSC (Diamond DSC, Perkin-Elmer, Waltham, MA, USA) and analyzed against a reference pan filled with air. The sample was tempered at 30 °C for 60 s and afterwards heated to 80 °C within 300 s corresponding to a heating rate of 10 °C/min. GT_O_ was calculated by the Pyris™ software (version: 10.1.0.0411, Perkin-Elmer, Waltham, MA, USA). All samples were analyzed in triplicate and the results were averaged.

### 2.3. Determination of Pasting Temperatures

Pasting temperatures of all samples were determined viscometrically using a Rapid-Visco-Analyzer (RVA, Perten Instruments, a Perkin-Elmer Company, Waltham, MA, USA) according to a malt specific procedure with optimized parameters, as described in detail elsewhere [16,33].

In short, 6.25 g of malt flour were mixed with 25 mL deionized water (based on a malt moisture basis of 5.0%) in an RVA sample can. Then, the following procedure (Table 1) was performed by the TCW3 software (version: 3.15.3.347, © Perten Instruments, a Perkin-Elmer Company, Waltham, MA, USA).

In this study, the pasting temperature which is calculated automatically by the TCW3 software is referred to as the unadjusted pasting temperature (PT_5_). To compensate for the described inaccuracies of this evaluation, the viscometric raw data of each measurement was used to recalculate the compensated pasting onset temperature [17], referred to in this research as (adjusted) pasting onset temperature PT_O_.

(1) $${\mathrm{PT}}_{O}=0.7253\cdot {\mathrm{PT}}_{5}+13.003$$

### 2.4. Micro-Mashing Trials

**Figure 1:**
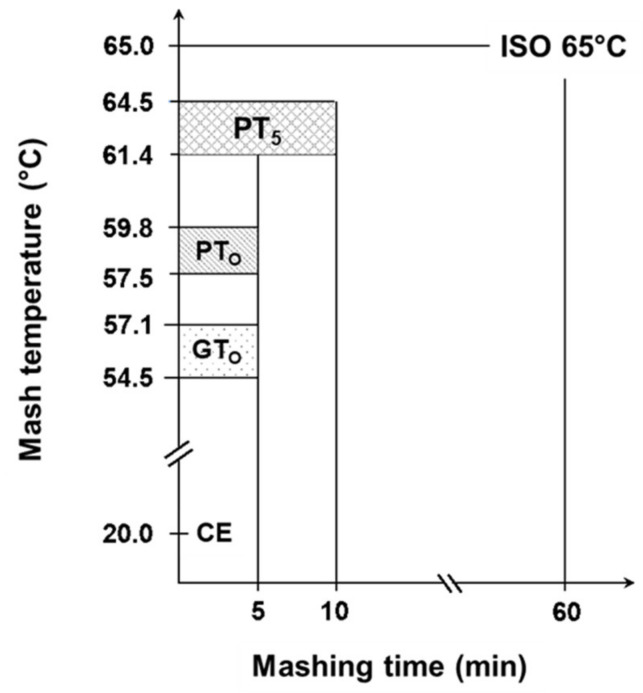
Overview of range of sample specific temperature levels during isothermal micro-mashing trials. Abbreviations: CE: cold extract, GT_O_: gelatinization onset temperature, PT_O_: pasting onset temperature, PT_5_: unadjusted pasting onset temperature, ISO 65 °C: laboratory standard method. Mashing time was 5 min for CE, GT_O_, PT_O_ and PT_5_, whereby PT_5_ was also mashed for 10 min. Mashing time for ISO 65 °C was 60 min.

Based on the determined gelatinization and pasting temperatures, nine malts being representative of the entire temperature range were selected for further trials. The samples were micro-mashed in the RVA at a malt-to-water ratio of 1:4 *w*/*w*. The sample mass was adjusted for its water content to equate to 6.25 g malt with a water content of 5% and 25 g of water. Each sample was tempered for 1 min at 50 °C, followed by rapid heating (0.2 °C/s) to the earlier determined gelatinization (GT_O_) or respective pasting onset temperatures (PT_O_), which was then held constant for 5 min. At the highest temperature level (PT_5_), all samples were mashed for 5 min or 10 min. To stop enzymatic degradation and preserve the present morphological status of the starch granules after mashing, samples were rapidly cooled by putting the RVA sample cans into an ice bath. In addition, 1 mL of 625 mM AgNO_3_-solution was added to inhibit ongoing amylase activity. For further analyses, samples were transferred to 50 mL centrifuge tubes and centrifuged (Rotina 420R, Andreas Hettich GmbH, Tuttlingen, Germany) at 4000 rpm for 5 min. In this study, these samples are referred to as enzymatic (E). To differentiate morphological from enzymatic effects, all enzymatic micro-mashing trials were repeated under inhibited conditions by replacing deionized water by an equivalent of AgNO_3_-solution (25 mM), which has been shown to completely inhibit α-amylase activity [16,34]. In this research these samples are referred to as inhibited (I). To determine the samples’ initial properties prior to thermal and/or enzymatic treatment, all malts were extracted at 20 °C for 5 min in presence of AgNO_3_ and centrifuged as described earlier. The results of these samples that are referred to as cold extract (CE), were set as a lower baseline for all further experiments. By contrast, the upper limit was investigated using a standardized laboratory method to determine the maximum malts’ extract content (R-207.00.002, [33]). To achieve this, mashing of the samples was performed isothermally at 65 °C for 60 min (ISO 65°C). The results of CE and ISO 65 °C mashing describe the range of initial malt properties and total morphological and enzymatic transformation and can thus be used to benchmark the process efficiency of different mashing temperatures. Figure 1 gives an overview of the applied temperature-time regimes.

### 2.5. Viscometric Analyses

During the micro-mashing trials in the RVA, the viscosity of the samples was permanently recorded and three viscometric characteristics were calculated. The initial viscosity (IV) of the unpasted mash was calculated by averaging the results during the tempering phase at 50 °C [17], where η_t_ is the viscosity determined every second.

(2) $$\mathrm{IV}=\frac{1}{36}\overset{60}{\underset{t=25}{\sum }}\eta_{t}$$

In addition, the peak viscosity (PV) refers to the highest viscosity detected during mashing, whereas the viscosity at the end of mashing is termed final viscosity (FV). In addition, the viscosity progression factor (VPF) describes the potential to increase viscosity during mashing:

(3) $$\mathrm{VPF}=\frac{\mathrm{PV}}{\mathrm{IV}}$$

### 2.6. Visualization of Samples

Starch granules were visualized by confocal laser scanning microscopy (CLSM, ECLIPSE Ti, 20× air objective, Nikon Instruments, New York, NY, USA. For this, 1 mL of the centrifuged suspension was homogenized and diluted with 25 mL deionized water. After homogenization, 1 mL of the resulting solution was mixed with 10 µL of staining solution (1% *w*/*w*, Light Green SF Yellowish, Merck KGaA, Darmstadt, Germany) to label impurities. After incubation in the dark for 5 min, 50 µL Safranin O solution (0.3% *w*/*w*, Safranin O, Merck KGaA, Germany) were added to counterstain starch granules. The mixture was incubated for 5 min and analyzed by CLSM using an argon ion laser (wavelength: 488 nm) and a diode laser (wavelength: 635 nm).

### 2.7. Particle Size Distribution of Starch Granules

Particle size distributions were determined by laser diffraction (Mastersizer 3000, Malvern Instruments GmbH, Malvern, UK). Prior to measurement, the centrifuged samples were homogenized by filling the tubes with deionized water and mixing the suspensions for 5 min. Sample material was added to the sample beaker of the Mastersizer until an obscuration of 3–20% was reached. Each sample was analyzed in triplicate and the results were averaged. To differentiate the effects of varying temperatures on A- and B-type granules, the data is presented in two different types of graphs:

- A and B-type, considering all granule diameters ranging from 0 μm to 100 µm.
- B-type, considering only granule diameters ranging from 0 μm to 10 µm.

Without this differentiation, swelling and enzymatic hydrolysis of smaller B-type granules would be overlaid by the disproportionate volume increase of larger A-type granules.

### 2.8. Fermentable Sugars

The total amount of fermentable sugars (i.e., glucose, fructose, sucrose, maltose and maltotriose) was determined using high performance liquid chromatography (HPLC; Dionex^®^ ICS 5000 employing 2 × 250 mm Dionex^®^ CarboPack, PA10 analytical separating columns and 2 × 250 mm Dionex^®^ CarboPack PA10 Guard precolumns, Dionex Softron GmbH, 82110, Germering, Germany). Therefore, 3 mL of the supernatant obtained after centrifugation were filtered through a 0.45 µm membrane and 1 mL of the filtrate was diluted with 50 mL deionized water. A total of 1 mL of dilute solution was transferred to an HPLC vial and analyzed using a flow rate of 0.25 mL/min.

The measurements were evaluated using the software ChromeleonTM 6.0 (Thermo Fisher Scientific, Waltham, MA, USA). The original amount of sugar in the micro-mashes was calculated by considering the dilution factor.

The sugar yield was calculated according to the following equation:

(4) $$\mathrm{Sugar}\;\mathrm{yield}=\frac{c\left(x\right)}{c\left(\mathrm{ISO}\;65\;{}^{\circ}C\right)}\times 100$$

where c(x) is the amount of fermentable sugar obtained after isothermal mashing for 5 min or 10 min at a certain temperature level x (i.e., CE, GT_O_, PT_O_ or PT_5_) and c(ISO 65 °C) refers to the upper reference limit achieved after 60 min at 65 °C.

### 2.9. Statistical Analysis

Two independent batches of each sample condition were prepared, and all samples were analyzed at least in duplicate. Thus, at least, a fourfold determination was carried out. Unless mentioned otherwise, Excel was used for data analysis. For the *t*-test, the software Statgraphics Centurion 18 was used.

## 3. Results and Discussion

### 3.1. Gelatinization and Pasting Onset Temperatures of Barley Malt Samples

**Figure 2:**
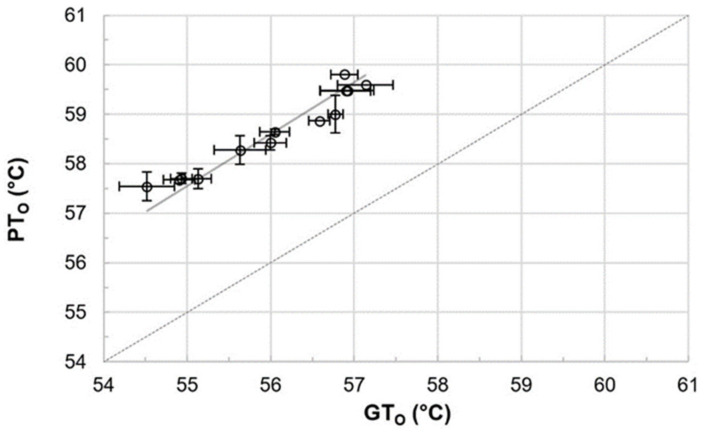
Comparison of and correlation between gelatinization onset (GT_O_) and pasting onset (PT_O_) temperatures (*r* = 0.97). Data points refer to mean values and error bars represent the standard deviation. The dotted line is the bisecting line.

Gelatinization and pasting onset temperatures were analyzed to determine the accurate mashing temperatures for the subsequent micro-mashing trials. Overall, gelatinization onset temperatures (GT_O_), which were obtained by DSC measurements, ranged from 54.5 to 57.1 °C (Figure 1), which are in a similar range as reported by others [12] for barley starch. It is important to highlight that these results did not overlap with the viscometrically determined pasting onset temperatures (PT_O_), ranging from 57.5 to 59.8 °C. On average, pasting onset temperatures of the samples were 2.6 °C higher than the respective gelatinization onset temperatures (Figure 2). The results point out that firstly, the gelatinization (M = 56.0 °C, SD = 0.92 °C, N = 12) and pasting onset temperatures (M = 58.6 °C, SD = 0.81 °C, N = 12) of each sample are significantly different (*p* < 0.001, two-tailed). Secondly, a linear correlation exists between both parameters (r = 0.97).

To the authors’ best knowledge, no correlation between gelatinization and pasting onset temperatures has been reported for barley malt samples, so far. For other cereals, a comparable behavior can be extracted from raw data published by Park et al. [35], who described the impact of amylose content and molecular weight of waxy and non-waxy rice starches on gelatinization and pasting onset temperatures. An analysis of the published gelatinization and pasting onset data also revealed a linear correlation (*r* = 0.99; results not shown). 

### 3.2. Impact of Different Temperature Levels on the Swelling and Hydrolysis of Starch Granules

#### 3.2.1. Cold Extract (20 °C)—Determination of the Initial Situation of Barley Malt Samples

**Figure 3:**
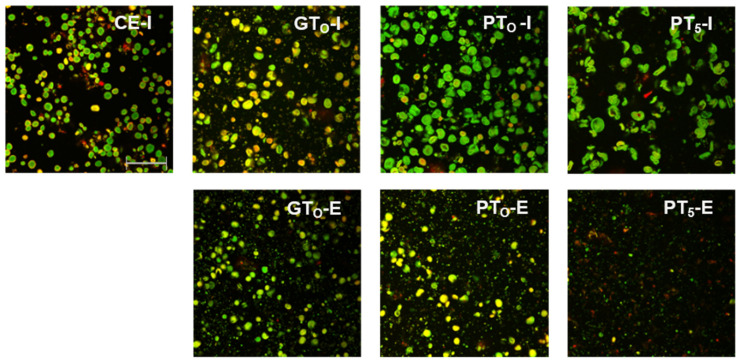
CLSM micrographs of inhibited (I) and enzymatic (E) barley malt samples after isothermal mashing for 5 min at sample specific temperatures levels. Abbreviations: CE: cold extract, GT_O_^:^ gelatinization onset temperature, PT_O_: pasting onset temperature, PT_5_: unadjusted pasting onset temperature. Scale bar refers to 200 µm.

**Figure 4:**
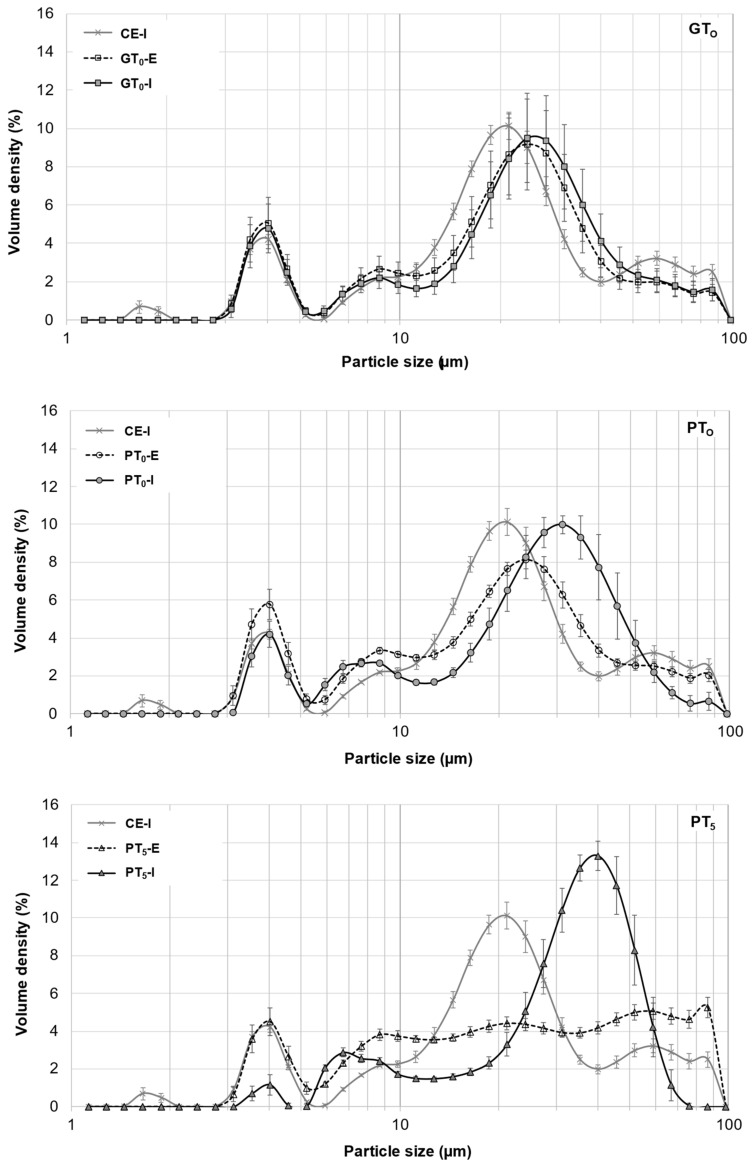
Averaged particle size distributions of A- and B-type granules after mashing for 5 min at GT_O_, PT_O_ and PT_5_. In all diagrams, CE-I is displayed as reference. Data points refer to mean values (*N* = 18) and error bars represent the standard deviation.

**Figure 5:**
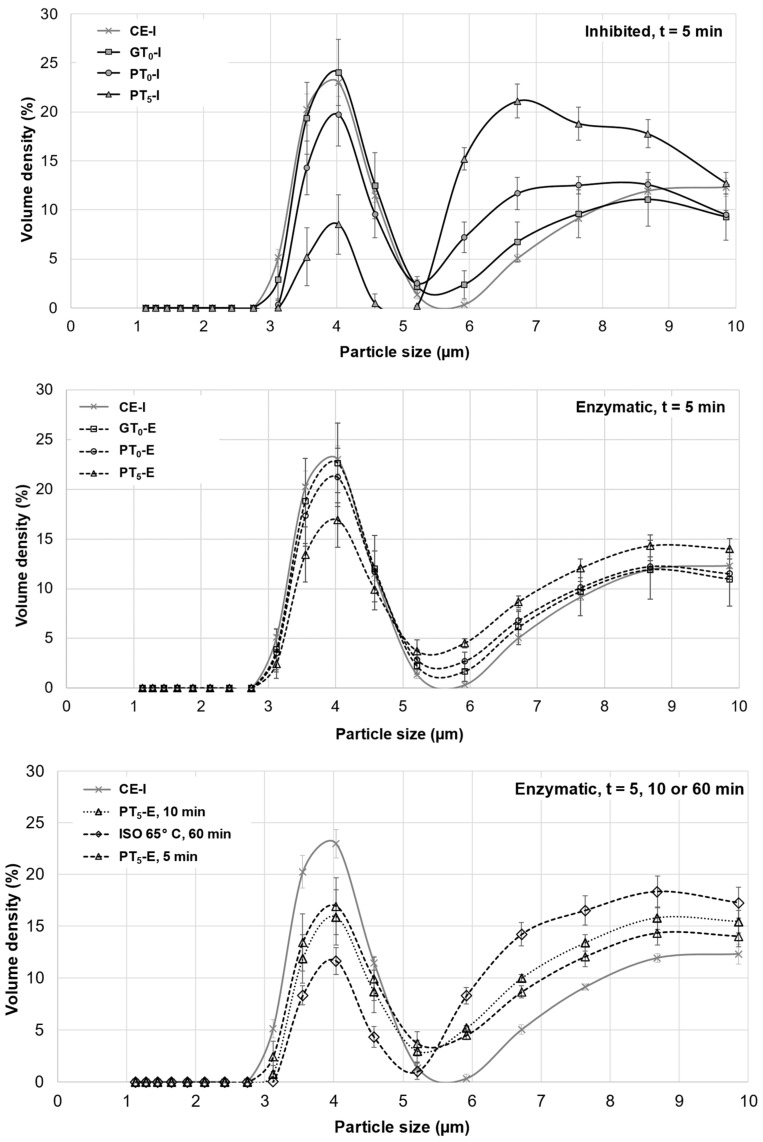
Averaged particle size distributions of B-type granules after mashing for 5 min at inhibited conditions, 5 min at enzymatic conditions and varying durations at enzymatic conditions. In all diagrams, CE-I is displayed as a reference. Data points refer to mean values (*N* = 18), error bars represent the standard deviation.

Starch granules from barley malt samples mashed at 20 °C under inhibited conditions (CE-I) exhibited a defined circular shape and no swelling was noticeable (Figure 3). Overall, the viscosity (Table 2) remained at a relative constant level and the particle size distribution (Figure 4) showed a barley starch-typical bimodal distribution with defined peaks around 4 µm and 20 µm for B-type and A-type granules, respectively [11,36]. For reasons of comparison, the particle size distribution of CE-I, which comprises the mean of nine barley malt samples, is shown as the lower baseline in all successive diagrams on particle size distributions (Figure 4 and Figure 5). 

**Figure 6:**
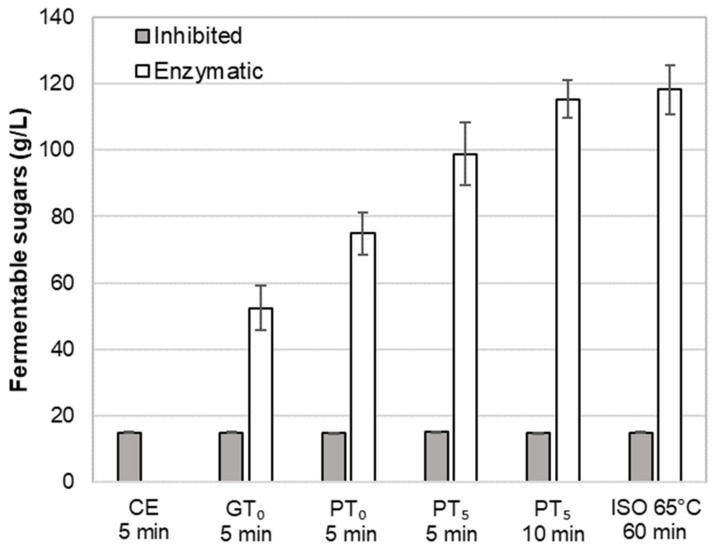
Impact of mashing at characteristics temperature levels for different durations at inhibited or enzymatic conditions on the average amount of fermentable sugars. Data points refer to mean values (*N* = 18) and error bars represent the standard deviation.

Regarding the total fermentable sugar content (Figure 6), on average, an amount of 15 g/L was determined in CE-I. This corresponds to an initial sugar content in the barley malt samples of 6.3% (dry basis), which is in accordance with previous investigations [37]. As enzymatic activity was inhibited for these cold extract trials, the sugars must be directly soluble in an aqueous phase. Compared to the maximum amount of fermentable sugars obtained by isothermal mashing (ISO 65°C, 118.3 g/L), the sugar yield of CE-I represents 12.4%.

#### 3.2.2. Impact of Mashing at Gelatinization Onset Temperature on Starch Granules

**Figure 7:**
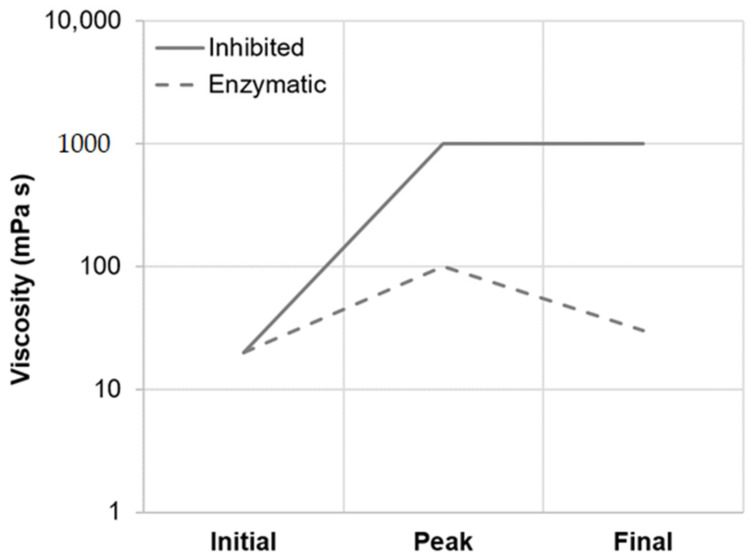
Principal viscosity progression of barley malt samples isothermally mashed at inhibited versus enzymatic conditions for 5 min. The curves are representative of all temperature levels investigated (i.e., GT_O_, PT_O_ and PT_5_).

As summarized in Table 2, viscometric data recorded during isothermal micro-mashing trials at the samples’ respective gelatinization onset temperatures (GT_O_, 54.5–57.1 °C; Figure 1) indicated an only marginal increase in viscosity at both inhibited and enzymatic conditions. In addition, at both conditions, the samples exhibited a similar mean viscosity progression factor (VPF) of 1.6. Nevertheless, a certain difference between inhibited and enzymatic conditions could be observed. As appears from Table 2 and the schematic representation in Figure 7, in contrast to enzymatic conditions, viscosity at inhibited conditions did not decrease after peaking, which is typical for viscometric analyses of malt [15]. The permanent increase in viscosity at this temperature level (i.e., GT_O_) can be explained by two different mechanisms as follows.

On the one hand, as indicated in Figure 4, the particle size distribution peak at 20 μm corresponding to A-type granules experienced a slight shift towards larger particle sizes, whereas the size of B-type granules remained unchanged at 4 μm. From this, it can be concluded that A-type granules unlike B-type granules underwent limited swelling, therefore contributing to the increase in the samples’ viscosities. As there was no significant difference between particle size distributions of inhibited and enzymatic samples, it was concluded that no significant enzymatic hydrolysis of swollen starch granules took place at GT_O_ resulting in consistent viscosities after the initial increase. On the other hand, an increased β-glucan solubility due to higher sample temperatures could also explain the slightly increased viscosities [38]. 

In this context, the limited swelling of starch granules could also be observed in CLSM micrographs (Figure 3). Although the borders of some granules seemed to blur, the circular shape was still present. This indicates that disruptive swelling had not occurred yet. As expected from the viscometric data and particle size distributions, the visual comparison of inhibited (GT_O_-I) and enzymatic (GT_O_-E) samples also did not reveal considerable differences in terms of morphology.

Regarding the amount of fermentable sugars (Figure 6), enzymatic mashes (GT_O_-E) contained, on average, 52.4 g/L. This represents a sugar yield of 43.2% and a clear increase as compared to the cold extract (CE-I). As expected, the average sugar level of inhibited mashes (GT_O_-I) was comparable to CE-I, which proves the successful inhibition of amylolytic enzymes.

As neither the viscosity progression nor the CLSM micrographs or particle size distributions indicated a massive loss of starch granules, the additional sugar determined for GT_O_-E samples must have originated from other sources. According to Sasaki and Matsuki [39] and Tester and Morrison [6], the quantity of leached amylose correlates with the extent of granule swelling, which itself is a function of the temperature. Therefore, it is possible that the observed non-disruptive granule swelling led to starch leaching followed by hydrolysis into fermentable sugars. Another conceivable mechanism could be based on increased enzymatic vulnerability of damaged starch granules either by enzymatic degradation during the malting process [18,40] or by mechanical damage during the milling process of the samples [41,42]. As damaged starch granules or fragments thereof undergo pasting and hydrolysis at lower temperatures [8], this could partially explain the increased amount of fermentable sugars of GT_O_-E samples compared to the results of CE-I.

#### 3.2.3. Impact of Mashing at Pasting Onset Temperatures on Starch Granules 

Mashing the samples at their respective pasting onset temperatures (PT_O_, 57.5–59.8 °C; Figure 1) led to significantly altered starch characteristics. Regarding the viscometric data given in Table 2, under inhibited conditions, the viscosity progression factor increased significantly (PT_O_-I: mean VPF = 4.1) as compared to gelatinization onset levels (GT_O_-I: mean VPF = 1.6). At enzymatic conditions the viscosity progression factor was reduced (PT_O_-E: mean VPF = 2.4). Taking in consideration that the viscosity decreased again during mashing by 31%, resulting in a final viscosity comparable to the level of GT_O_-E, it can be concluded that at PT_O_-E, granule swelling is induced and followed by partial hydrolysis. This theory is further supported by the particle size distributions shown in Figure 4. Under inhibited conditions (PT_O_-I), the A-granule peak shifted from about 20 to 31 µm indicating the generation of larger granules due to swelling. By contrast, under enzymatic conditions (PT_O_-E), this A-granule peak was significantly reduced in volume density and particle size, indicating that a sizeable proportion of the swollen granules had been hydrolyzed after swelling.

To better visualize and distinguish the effect of mashing temperature levels at inhibited and enzymatic conditions on B-type granules, respectively, their averaged particle size distributions are focused on in Figure 5. In contrast to mashing at GT_O_, a limited but significant shift from the major 3–5 µm fraction to 6–9 µm was noticeable under inhibited conditions (PT_O_-I). Under enzymatic conditions (PT_O_-E), this shift seemed to be reversible, suggesting that a limited number of B-type granules can undergo swelling and successive hydrolysis at this temperature level. Compared to A-type granules, the extent of swelling and hydrolysis of B-type granules, however, seemed to be limited at PT_O_, which is in accordance with findings from other researchers [43].

CLSM micrographs (Figure 3) support this assumption as starch granules of inhibited samples (PT_O_-I) were significantly swollen, implying increased granule sizes and reduced circular shapes. After mashing at enzymatic conditions (PT_O_-E), a reduction of visible granules as compared to the inhibited samples (PT_O_-I) was noticeable.

In addition, the observed swelling and hydrolysis of starch granules at PT_O_-E was also reflected in an increased amount of fermentable sugars of, on average, 74.9 g/L representing a sugar yield of 61.7% (Figure 6). 

#### 3.2.4. Impact of Mashing at Unadjusted Pasting Onset Temperatures on Starch Conversion

Unadjusted pasting temperatures (PT_5_) were derived from RVA measurements without temperature compensation and ranged from 61.4 to 64.5 °C (Figure 1). 

Under inhibited conditions (PT_5_-I), mashing at this temperature level led to intense pasting reflected by a viscosity progression factor of 96.5 (Table 2) and the formation of a thick paste. The initial diameter of the A-type granules nearly doubled from about 20 μm (CE-I) to around 40 µm (PT_5_-I), reflecting the intensity of granule swelling (Figure 4) as well as an increase in viscosity for inhibited samples. As indicated by the shift and broadening of the first peak from 3–5 µm (CE-I) to 5–10 µm (PT_5_-I), this time, also B-type granules underwent noticeable swelling (Figure 5). This intense granule swelling including loss of circularity and integrity could also be observed in CLSM micrographs (Figure 3).

Under enzymatic conditions (PT_5_-E), the viscometric characterization revealed the typical increase in viscosity (PV) and subsequent reduction towards the initial level (FV ≈ IV) (Table 2, Figure 7). In addition, a comparatively high mean VPF of 5.2 was detected (Table 2). As shown in Figure 4, the cumulated particle size distribution no longer exhibited an A-granule peak, neither of the initial, nor of the swollen state. This speaks in favor of intensive hydrolysis of A-type granules at this temperature level. In contrast, swollen B-type granules were only partially degraded (Figure 5) and approximately 75% of the B-type granules were still present in their native state. This result was also reflected in CLSM micrographs, indicating that swollen A-type granules disappeared after mashing, whereas B-type granules were still visible (Figure 3). 

In terms of fermentable sugars, on average, 98.8 g/L were detected in the samples, reflecting a sugar yield of 83.5% (Figure 6). Compared to mashing at pasting onset temperature levels, the sugar yield increased by 23.9%, which can be mainly explained by the complete disruption of A-type granules. 

#### 3.2.5. Forced Pasting Due to Prolonged Mashing and Increased Temperatures

In order to evaluate the impact of a prolonged mashing on the remaining B-type granules after mashing for 5 min at unadjusted pasting temperatures (PT_5_, 5 min) (see Section 3.2.4), the mashing time at PT_5_ was extended to 10 min (PT_5_, 10 min). Interestingly, no significant effect of mashing time on B-type granule particle size distribution was observed (Figure 5) but the amount of fermentable sugars increased significantly to, on average, 115.3 g/L, representing a sugar yield of 97.5% (Figure 6). 

These results could indicate that mashing at PT_5_ for ≤5 min led to rapid starch pasting and endo-enzymatic hydrolysis of viscosity-increasing starch fragments, while exo-enzymatic hydrolysis of starch into fermentable sugars seemed to require more time (5 min < x ≤ 10 min). As mentioned before, the prolonged mashing did not lead to a further swelling and saccharification of B-type granules. Thus, the increased amount of fermentable sugars was not linked to macroscopic structural changes of B-type granules. Overall, the obtained amount of fermentable sugars lies in the range of results of other research work [43]. However, the conversion rates in these studies were significantly lower, which could be explained by the application of sample unspecific, preset temperature levels during mashing.

For the determination of the overall upper limit, barley malt samples were isothermally mashed at 65 °C for 60 min (ISO 65°C) according to the MEBAK standard procedure (see Section 2.4). This resulted in an additional, but still not entire degradation of B-type granules. As illustrated in Figure 5, the initial mass percentage of B-type granules was reduced by approximately 50%. Moreover, the averaged amount of fermentable sugars increased to 118.3 g/L, corresponding to a sugar yield of 100% (Figure 6). As compared to the results obtained after mashing for 10 min at PT_5_, this limited increase can be explained by the minor contribution of B-type granules to the total starch balance [11] and the remaining, unpasted B-type granules.

## 4. Conclusions

Overall, the present investigation points out the importance and advantage of a sample specific initial mashing temperature to achieve a rapid and comprehensive starch conversion.

As mashing at gelatinization onset temperatures (GT_O_) led to only limited swelling of A-type and no swelling of B-type granules, this temperature level does not seem to be a relevant process parameter for the mashing process. Although pasting onset temperatures (PT_O_) were, on average, only 2.6 °C higher than respective GT_O_, mashing at this temperature level induced significant A-type granule swelling followed by amylolytic hydrolysis. Therefore, PT_O_ can be seen as the beginning of starch pasting and therefore be referred to as a minimal required initial mashing temperature. However, this temperature level is not sufficient to completely paste and hydrolyze A and B-type granules.

The application of the unadjusted pasting onset temperature (PT_5_), determined by the current viscometric standard analyzing procedure, leads to complete A-type and partial B-type granule conversion, and thus a sugar yield of 97.5% after only 10 min of mashing. Therefore, the application as initial mashing temperature seems to be suitable to balance rapid starch pasting as well as preservation of amylolytic enzymes, thus resulting in time- and energy-optimized starch conversion. 

In view of transferring these results to industrial processes, however, it needs to be kept in mind that (1) all samples were modern spring barley varieties that were malted intensively at lab scale resulting in high amylolytic activities, (2) the malt samples were fine-milled by a laboratory disc-mill and (3) PT_5_ of the samples did not exceed 65 °C. Therefore, the impact of malting intensity, especially regarding the resulting amylolytic potential as well as the applied physical modification during malt milling should be investigated in the future. In this research work, applying unadjusted pasting temperatures always resulted in higher sugar yields compared to samples mashed at pasting onset temperatures. Prospective investigations should also focus on the existence of an upper temperature limit, which must not be exceeded to avoid rapid amylase inactivation.

In addition to the starting point of pasting, which was investigated in this study, also the appropriate final pasting temperature should be assessed in future research. As this would allow to hydrolyze starch of temperature resistant B-type granules, this knowledge could contribute to further increasing the sugar yield and conversion efficiency during mashing.

Overall, the results speak in favor of applying a raw material adapted initial mashing temperature and point out that it is important to clearly differentiate between the terms gelatinization and pasting of malt starch. As saccharification requires intense starch granule swelling followed by disruption and amylolytic degradation, only the term pasting is appropriate in this context.

## Figures and Tables

**Table 1:** Temperature-time regime and pre-setting of the analysis procedure.

Time (mm:ss)	Type	Value
00:00	Temp	50 °C
00:00	Speed	960 rpm
00:10	Speed	160 rpm
01:00	Temp	50 °C
04:42	Temp	95 °C
06:00	Temp.	50 °C
End of test time:	06:30 (mm:ss)	
Initial idle temperature:	50 ± 1 °C	
Time between readings:	1 s	

**Table 2:** Impact of micro-mashing on the evolution of viscometric characteristics (IV initial viscosity, PV peak viscosity, FV final viscosity, VPF viscosity progression factor) of barley malt samples: CE cold extract, GT_O_ gelatinization onset temperature, PT_O_ pasting onset temperature, PT_5_ unadjusted pasting onset temperature. Reported values refer to the range as well as mean (M) viscosities of the different samples (*N* = 18) at the specified temperature levels.

Sample Condition	Temperature Regime	IV (mPa s)	PV (mPa s)	FV (mPa s)	Mean VPF
Inhibited (I)	CE	17–22 (M = 20)	20–26 (M = 24)	19–24 (M = 22)	1.2
	GTO	21–26 (M = 24)	35–44 (M = 38)	35–42 (M = 37)	1.6
	PTO	22–26 (M = 24)	59–211 (M = 97)	53–211 (M = 96)	4.1
	PT5	22–25 (M = 23)	1242–3407 (M = 2227)	1252–3407 (M = 2239)	96.5
Enzymatic (E)	GTO	19–25 (M = 22)	32–37 (M = 36)	29–35 (M = 33.0)	1.6
	PTO	20–24 (M = 22)	43–68 (M = 51)	31–39 (M = 35)	2.4
	PT5	20–26 (M = 22)	96–122 (M = 111)	26–34 (M = 30)	5.2

## Data Availability

Data will be made available upon request to the corresponding author.
